# Novel Toolboxes for the Investigation of Activity-Dependent Myelination in the Central Nervous System

**DOI:** 10.3389/fncel.2021.769809

**Published:** 2021-11-02

**Authors:** Jack Kent Heflin, Wenjing Sun

**Affiliations:** Department of Neuroscience, Wexner Medical Center, The Ohio State University, Columbus, OH, United States

**Keywords:** activity-dependent myelination, local stimulation, AMPA receptor, glutamate uncaging, time-lapse imaging

## Abstract

Myelination is essential for signal processing within neural networks. Emerging data suggest that neuronal activity positively instructs myelin development and myelin adaptation during adulthood. However, the underlying mechanisms controlling activity-dependent myelination have not been fully elucidated. Myelination is a multi-step process that involves the proliferation and differentiation of oligodendrocyte precursor cells followed by the initial contact and ensheathment of axons by mature oligodendrocytes. Conventional end-point studies rarely capture the dynamic interaction between neurons and oligodendrocyte lineage cells spanning such a long temporal window. Given that such interactions and downstream signaling cascades are likely to occur within fine cellular processes of oligodendrocytes and their precursor cells, overcoming spatial resolution limitations represents another technical hurdle in the field. In this mini-review, we discuss how advanced genetic, cutting-edge imaging, and electrophysiological approaches enable us to investigate neuron-oligodendrocyte lineage cell interaction and myelination with both temporal and spatial precision.

## Introduction

Myelination refers to the process of wrapping axons with compact layers of the plasma membrane, and it occurs primarily postnatally (Foran and Peterson, 1992; Baumann and Pham-Dinh, 2001; Nishiyama et al., 2009). The presence of myelin sheaths drastically increases the conduction velocity along axons. As the conduction velocity can be fine-tuned by myelin thickness and sheath length (Ford et al., 2015; Etxeberria et al., 2016), myelin is also pivotal in maintaining precise spatiotemporal activity patterns in neuronal circuits. Several recent studies particularly highlighted the importance of myelination for proper neuronal circuit activities and cognitive function in health and diseases (Pan et al., 2020; Steadman et al., 2020; Chen et al., 2021). Myelin sheaths in the central nervous system (CNS) are exclusively formed by oligodendrocytes that are generated from the differentiation of oligodendrocyte precursor cells (OPCs). Oligodendrocytes have the innate capability to wrap fibrous structures (Lee et al., 2012; Bechler et al., 2015). With the development of various pharmacological and genetic tools, it became clear that oligodendrogenesis and myelin formation are regulated by both intrinsic programs and extrinsic factors (Li and Richardson, 2016; Mayoral and Chan, 2016; Fletcher et al., 2018). Accumulating evidence has shown that social interaction (Liu et al., 2012; Makinodan et al., 2012), experience (Hughes et al., 2018), sensory inputs (Mangin et al., 2012; Hill et al., 2014), and motor learning activities (McKenzie et al., 2014; Bacmeister et al., 2020) modulate myelin adaptation and oligodendrocyte lineage cell behavior. Recent studies using state-of-the-art optogenetic (Gibson et al., 2014) and chemogenetic (Mitew et al., 2018) techniques showed that stimulating neuronal firing increased myelin thickness along the axons and oligodendrogenesis within the stimulated region. However, our understanding of the mechanisms underlying “activity-dependent myelination” is still limited.

For the past two decades, the development of various transgenic fluorescence reporter mouse lines has enabled us to directly visualize oligodendrocyte lineage cells at different stages (Mallon et al., 2002; Yuan et al., 2002; Karram et al., 2008; Zhu et al., 2008; Hughes et al., 2013). Genetic fate mapping studies using OPC specific Cre or CreER lines crossed with Cre-reporter mice have also generated vast information regarding oligodendrocyte lineage plasticity in both physiological and pathological conditions across multiple CNS regions (Lappe-Siefke et al., 2003; Rivers et al., 2008; Kang et al., 2010; Zhu et al., 2011; Tognatta et al., 2017; Huang et al., 2019). More recent studies have focused on manipulating the oligodendroglial-specific expression of key molecules in regulating oligodendroglial lineage plasticity or neuron-oligodendroglial communication. Together with the development of novel electrophysiological and optical techniques, we now have more comprehensive tools to study the underlying mechanisms for activity-dependent myelination.

## Manipulating The Expression of Postsynaptic Neurotransmitter Receptors in Oligodendrocyte Lineage Cells

The discovery of neuron-OPC synaptic contacts (Bergles et al., 2000) has inspired the myelin research field for the last 2 decades. The prevalence of such synapses across different CNS regions (Lin and Bergles, 2004; Jabs et al., 2005; Lin et al., 2005; Ge et al., 2006; Káradóttir et al., 2008; Kukley et al., 2008; Mangin et al., 2008; Vélez-Fort et al., 2010), including the white matter (Kukley et al., 2007; Ziskin et al., 2007), and the fact that synaptic contacts enable neuron-OPC communication with both temporal and spatial precision position them as an ideal candidate to instruct activity-dependent myelination. Novel genetic mouse models that specifically silence or overexpress different postsynaptic neurotransmitter receptors in oligodendrocyte lineage cells can manipulate neuron-OPC synaptic transmission without interfering with neuron-neuron communication, thereby providing in-depth insights into the functional implications of these neuron-OPC synapses.

**Figure 1:**
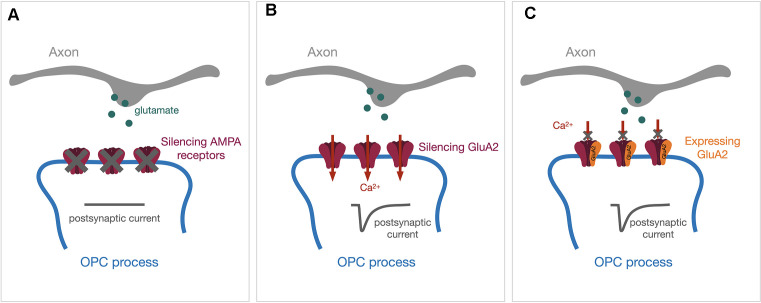
Genetic approaches to manipulate the expression of AMPA receptors in OPCs. Synaptic vesicles containing glutamate (green dots) will be released upon the arrival of action potentials. The release of glutamate will activate post-synaptic AMPA receptors and evoke a post-synaptic current (dark gray traces). **(A)** Genetically silencing all AMPA receptor subunits will diminish AMPA receptor-mediated postsynaptic currents. **(B)** Silencing the GluA2 subunit alone will increase Ca^2+^ permeability through AMPA receptors. **(C)** Expressing the GluA2-containing AMPA receptor will block the Ca^2+^ entry through AMPA receptors. OPCs, oligodendrocyte precursor cells.

Glutamatergic postsynaptic currents in OPCs exhibit fast kinetics and are primarily mediated through AMPA receptors. In a recent study, Kougioumtzidou et al. (2017) created several murine transgenic lines to reduce or eliminate AMPA receptor-mediated postsynaptic responses by crossing Sox10-Cre line and Rosa-YFP reporter line with different combinations of Gria2^flox/flox^ line, Gria4^flox/flox^ line, and Gria3 null line. Triple knock-out of GluA2–4 showed almost no AMPA receptor-mediated spontaneous synaptic responses (Figure 1A) and exhibited reduced numbers of mature oligodendrocytes within the corpus callosum in mice at both P14 and P53, while the proliferation of OPCs remained unaltered (Kougioumtzidou et al., 2017). Similar results in oligodendrocyte lineage were observed with Gria2^−/−^Gria3 null double transgenic mice, where the AMPA receptor-mediated currents were reduced by half (Kougioumtzidou et al., 2017). However, neither single transgenic Sox10-Cre: Gria2^flox/flox^ nor germline Gria3 null mice alone displayed any phenotype in oligodendrocyte lineage development and myelination, suggesting that there might be compensatory mechanisms between the expression of AMPA receptor subunits (Kougioumtzidou et al., 2017).

Among GluA1–4 subunits, GluA2 determines the Ca^2+^ permeability such that only receptors lacking GluA2 are Ca^2+^ permeable. Previous studies demonstrated that OPCs express Ca^2+^ permeable AMPA receptors in the hippocampus (Bergles et al., 2000; Ge et al., 2006). In the corpus callosum, Ca^2+^ permeable AMPA receptors can only be detected in OPCs during adulthood (Ziskin et al., 2007), but not in the early postnatal period (Kukley et al., 2007). One emerging question is whether Ca^2+^ permeability through AMPA receptors regulates oligodendrocyte lineage plasticity and activity-dependent myelination. Different genetic strategies have been utilized to address this question. In the study mentioned above, Kougioumtzidou et al. (2017) did not observe any changes in oligodendrocyte lineage development and myelination from the corpus callosum at P14 using the Sox10-Cre: Gria2^flox/flox^ line to increase Ca^2+^ permeability by deleting GluA2 in OPCs (Figure 1B). Conversely, Khawaja et al. (2021) recently generated an inducible GluA2 overexpression line (Sox10-CreER:Ai14:R26-Gria2) where the decreases in Ca^2+^ permeability through AMPA receptors (Figure 1C) resulted in an increase in OPCs only in the adult mice. Myelination in younger mice from the same line was not affected by GluA2 overexpression when 4-Hydroxytamoxifen was injected early postnatally (Khawaja et al., 2021).

Besides transgenic mouse lines, viral gene delivery approaches are routinely used for transgene expression *in vivo*. Although most adeno-associated viruses (AAV) serotypes exhibit dominance in neuronal tropism with constitutive promoters driving the gene expression (Burger et al., 2004; Cearley and Wolfe, 2006), several studies successfully drove oligodendrocyte-specific gene expression *in vivo* using AAVs with oligodendrocyte-specific promoters, such as myelin basic protein or myelin-associated glycoprotein (Chen et al., 1998; Lawlor et al., 2009; Von Jonquieres et al., 2016), or packaging AAV vectors with a novel oligotropic capsid (Powell et al., 2016; Weinberg et al., 2017). However, so far, AAV particles rarely transduce OPCs in a specific and consistent fashion *in vivo*. Instead, the retrovirus is a good alternative as it only transfects dividing cells (Van Praag et al., 2002), and OPCs are the major source of proliferating cells in the postnatal CNS (Dawson et al., 2003). While also aiming at manipulating AMPA receptor-dependent Ca^2+^ permeability, Chen et al. (2018) modified OPC-expressed AMPA receptors *in vivo* using retroviral vectors early postnatally. They delivered retroviral vectors expressing either full-length GluA2 with point mutations or truncated GluA2 into the corpus callosum during postnatal 2–3 weeks and reported that ~95% of transduced cells belong to oligodendrocyte lineage (Chen et al., 2018). This study showed that GluA2 point mutations increasing the AMPA receptor Ca^2+^ permeability enhanced OPC proliferation and suppressed their differentiation. Perturbing the trafficking of GluA2-containing AMPARs by expressing the cytoplasmic C-terminal of the GluA2 subunit decreased the OPC differentiation without affecting the proliferation during myelin development (Chen et al., 2018). While the two studies mentioned above used transgenic lines to manipulate GluA2 expression in the total OPC population, this study only targeted proliferating OPCs, which may explain the difference in their observations.

OPCs gradually lose synaptic contacts with neurons during their differentiation into oligodendrocytes, accompanied by a downregulation of ionotropic glutamate receptors (De Biase et al., 2010; Kukley et al., 2010). Nevertheless, Evonuk et al. (2020) crossed the PLP-CreER line with Gria^4tm1Mony^ mice (Fuchs et al., 2007) to specifically knock-out GluA4 in more mature PLP^+^ oligodendroglial cells. They reported that deleting GluA4 ameliorated myelin degeneration and axonal damage in a mouse model of multiple sclerosis (Evonuk et al., 2020). Oligodendrocyte lineage cells also express NMDA receptors (Káradóttir et al., 2005, 2008; Salter and Fern, 2005; Micu et al., 2006). These NMDA receptors are reported to be involved in mediating myelin responses after CNS injuries (Káradóttir et al., 2005; Micu et al., 2006, 2007). It has been suggested that remyelination after white matter injury is NMDA receptor-dependent (Lundgaard et al., 2013). Genetic models that specifically delete NMDA receptor subunit NR1 in OPCs exhibited normal myelination and did not show obvious alteration in survival or proliferation of OPCs (De Biase et al., 2011; Guo et al., 2012). However, Saab et al. (2016) recently reported that oligodendroglial NMDA receptors regulate GLUT1-mediated glucose import into the myelin compartment, and the deletion of NR1 in CNP^+^ oligodendroglial cells led to a delay in myelin development.

GABAergic interneuron-OPC synapses primarily activate Cl^−^-permeable GABA_A_ receptors in OPCs. Instead of hyperpolarizing the membrane potential, the activation of GABA_A_ receptor depolarizes the post-synaptic membrane due to high intracellular Cl^−^ concentration (Lin and Bergles, 2004; Passlick et al., 2013). GABAergic synaptic inputs in OPCs are therefore not electrically “inhibitory”. While direct GABAergic synaptic contacts in OPCs persist through the development to adulthood in the mouse hippocampus (Lin and Bergles, 2004), in the cortex those direct synaptic activities start decreasing after the second postnatal week and gradually switch to the volume transmission mode mediated by the extra-synaptic activation of GABA_A_ receptors (Vélez-Fort et al., 2010). In the neocortex, OPCs are highly connected with fast-spiking parvalbumin (PV) interneurons, and γ2 subunits of GABA_A_ receptors are exclusively expressed at post-synaptic sites of PV interneuron-OPC synapses (Orduz et al., 2015). Balia et al. (2017) bred NG2creERT2:GCamp3: γ2^fl/fl^ mice to specifically inactivate γ2- GABA_A_ receptor-mediated synapses in OPCs early postnatally. They reported no alteration in OPC proliferation or differentiation, but a decrease in OPC density at P30 compared to control mice, suggesting that γ2- GABA_A_ receptors play a role in OPC self-maintenance (Balia et al., 2017). In a more recent study from the same group, Benamer et al. (2020) further characterized this transgenic line and revealed that γ2 mutant mice exhibited abnormal axonal morphology and myelin distribution in PV interneurons, along with increased nodal and internodal lengths of myelinated PV axons. Those observed myelin defects decreased the firing frequency of PV interneurons and their connectivity with excitatory neurons, leading to the impairment of sensory function in γ2 mutant mice (Benamer et al., 2020). It will be interesting for future studies to investigate the functional role of synaptic contacts between other types of interneurons and OPCs, perhaps with genetic models that specifically delete GABA_A_ receptors in OPCs in general.

## Eliciting Local Postsynaptic Responses within OPC Processes

**Figure 2:**
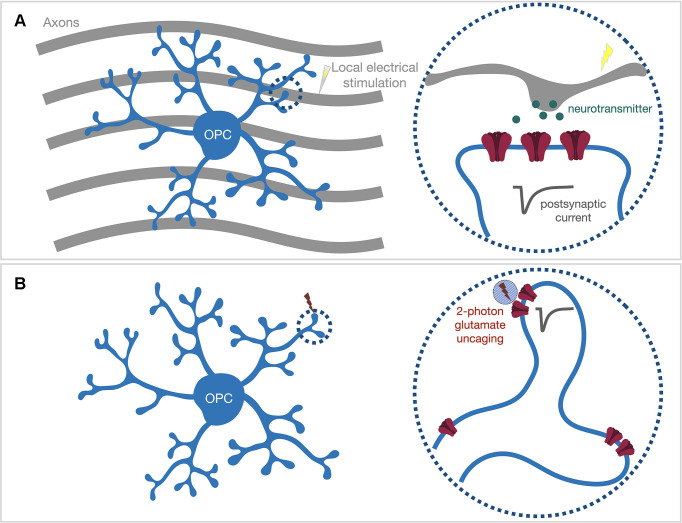
Locally activate postsynaptic receptors within OPC processes *via* either **(A)** local electrical stimulation or **(B)** 2-photon glutamate uncaging. **(A)** The mono-polar stimulating electrode can be placed in close proximity (~5 μm) to the target OPC process (dash circle) to only stimulate the axons passing the process segment. **(B)** Acute brain slices are bathed with caged-glutamate containing bath solution. The 2-photon uncaging laser (~720 nm) is pointed to the target locations by galvo scan mirrors inside the scan head. A brief uncaging laser pulse (0.6–1 ms) will uncage the caged-glutamate compound within the 2-photon excitation volume (blue shaded circle) and evoke an electrical response from post-synaptic neurotransmitter receptors (dark gray trace).

Accumulating data have suggested that neuronal activity-induced downstream signaling often occurs locally within the thin processes of oligodendroglial cells. Axonal stimulation using electrical, optogenetic, or chemogenetic strategies has been shown to induce local myelin basic protein synthesis within OPC processes (Wake et al., 2011) and increase myelin thickness of stimulated axons (Gibson et al., 2014; Mitew et al., 2018). Confocal time-lapse imaging studies have demonstrated that cellular processes of oligodendrocyte lineage cells actively contact and form initial ensheathment with axons, but the stabilization and elongation of formed myelin sheaths are regulated by neuronal activity (Hines et al., 2015; Mensch et al., 2015; Koudelka et al., 2016). Thus far, it is not clear whether the single contacted axon’s activity is sufficient to trigger downstream signaling cascades within OPC processes locally, or whether activities from a large group of axons are required to initiate global signaling that spreads out to the whole OPC process architecture. To tease apart these two scenarios, we need to use experimental paradigms that will only activate one or just a few synapses within a spatially confined region. We previously applied local electrical stimulation by placing a mono-polar stimulating electrode approximately 5 μm away from the target OPC process segment (Figure 2A; Sun et al., 2016). When combining electrophysiology recording with 2-photon Ca^2+^ imaging, we observed that such a local stimulus is sufficient to trigger Ca^2+^ entry within the local compartment with similar kinetics as the global voltage-gated Ca^2+^ channel (VGCC)-mediated Ca^2+^ entry induced by current injection (Sun et al., 2016). The somatic depolarization upon local stimulation was well below the activation threshold for VGCCs, and we did not observe a widespread global Ca^2+^ signal upon local stimulation. Our results suggest that only a limited number of axons passing the target OPC process were activated upon local stimulation, and also indicate a substantial attenuation along the OPC processes, as suggested by our previous theoretical simulation (Sun and Dietrich, 2013). Our observation also suggests the expression of voltage-gated ion channels within OPC processes (Sun et al., 2016). Therefore, the local synaptic response can be amplified or dampened through the activation of ion channels within processes. Since myelin basic protein, and likely other myelin proteins could be synthesized within the OPC processes (Wake et al., 2011), future studies could utilize local electrical stimulation together with advanced *in situ* hybridization techniques, such as RNAscope (Wang et al., 2012), to address remaining questions like whether local synaptic inputs are sufficient to initialize the transport, local expression, and translation of myelin-specific mRNAs.

Nevertheless, the spatial resolution of local electrical stimulation is not sufficient to accurately identify which nearby synapses are activated, especially when there is no optical tool available to visualize where synapses are located along OPC processes. To circumvent this limitation, the alternative approach is to optically activate postsynaptic neurotransmitter receptors on OPC processes with high optical resolution (Figure 2B). 2-photon glutamate uncaging generates an optically controlled transient release of glutamate by brief photolysis of caged-glutamate within the diffraction-limited 2-photon excitation volume (Matsuzaki et al., 2001). A single uncaging pulse applied at the dendritic spine head of neurons can produce an AMPA receptor-mediated current response similar to a synaptic response (Matsuzaki et al., 2001). We reported that the same strategy could be used to map AMPA receptors in OPCs (Sun et al., 2016). Although OPC processes are not equipped with spine structures that clearly indicate the clustering of AMPA receptors, we demonstrated that AMPA receptors are expressed along the process (Sun et al., 2016). The fact that the same uncaging pulse (720 nm, 0.65 ms, ~35–45 mW at the surface of the specimen; Sun et al., 2016) produced variable response sizes along a process segment suggests that AMPA receptors do not distribute homogeneously, but instead, might cluster in “hot spots”. However, whether those AMPA receptor “hot spots” represent locations of neuron-OPC synapses needs further investigation.

Beyond mapping AMPA receptor distribution, 2-photon glutamate uncaging may be useful to explore the functional implication of neuron-OPC synapses. For example, it can be used to study how OPCs integrate neuronal inputs from different synaptic contact sites and initialize downstream signaling cascades. Various electrophysiological studies characterized different voltage-gated ion channel expression in OPCs (Steinhäuser et al., 1994; Kressin et al., 1995; Akopian et al., 1996; Chittajallu et al., 2004; Tong et al., 2009; De Biase et al., 2010; Kukley et al., 2010; Haberlandt et al., 2011), and many of those ion channels, e.g., calcium and potassium channels, are likely expressed on the membrane of thin processes (Sun et al., 2016). The fact that voltage signals strongly attenuate along the OPC process (Sun and Dietrich, 2013) highlights the importance of understanding how these locally expressed ion channels respond to inputs from multiple synapses and shape the post-synaptic membrane potentials within OPC process compartments. The size of integrated local post-synaptic membrane potential might determine whether downstream signaling cascades can be triggered, e.g., whether Ca^2+^ channels can be activated and elicit a local Ca^2+^ transient. While local electrical stimulation cannot accurately determine how many synapses will be activated and where those activated synapses are located, 2-photon uncaging pulses can be pointed to multiple spatially apart spots with a minimal switching time (0.6–1 ms per uncaging pulse plus 0.1 ms laser switching time; Sun et al., 2016). Therefore, when combined with Ca^2+^ imaging and patch-clamp recording, 2-photon uncaging is an ideal tool to investigate the voltage integration of synaptic inputs from different sites and the subsequent Ca^2+^ signaling within a local process compartment. Glutamate uncaging is also suited to study activity-dependent synaptic or morphological plasticity. Ge et al. (2006) reported Ca^2+^ permeable AMPA receptor-mediated long-term potentiation (persistent strengthening of synaptic transmission) in OPCs from the murine hippocampus. Thus far, it is unclear whether such plasticity and potentially other types of synaptic plasticity exist at an individual synapse level and whether those types of synaptic plasticity are required for local translation of myelin proteins. Additionally, it is known that OPCs are motile and have filopodia-like protrusions constantly extending and retracting (Hughes et al., 2013). Understanding whether synaptic inputs can guide those dynamic protrusion movements to form initial contact with axons will be an important direction for future investigations. Indeed, a similar approach has been used in neuronal studies to demonstrate the occurrence of long-term potentiation at individual spines (Matsuzaki et al., 2004) and repetitive uncaging pulses-induced formation of new functional spines (Kwon and Sabatini, 2011). Taken together, 2-photon glutamate uncaging combined with other techniques is a powerful tool to tackle important questions like whether local synaptic integration is functionally meaningful and whether any specific neuronal activity patterns are preferred for initiation of myelin wrapping.

## Capturing The Dynamics of The Myelin Formation with Time-Lapse Imaging

Myelination is a multi-step process that involves the proliferation and differentiation of oligodendrocyte precursor cells followed by the initial contact and ensheathment of axons by mature oligodendrocytes. The whole process spans a long temporal window, and conventional end-point studies cannot capture the dynamics during the whole process. Recent studies have employed time-lapse imaging techniques based on confocal microscopy in the zebrafish model and generated crucial information about the initial myelin sheath formation along axons (Hines et al., 2015; Mensch et al., 2015; Koudelka et al., 2016). Those studies changed our previous knowledge or assumption that oligodendrocytes only select the electrically active axons to make the initial contact and subsequently start the wrapping process to form a sheath segment. Instead, oligodendrocytes constantly make contacts with axons and form initial myelin sheath segments, but at the same time, also often retract some formed sheaths. Therefore, the stabilization of those established sheaths is the key, and this step is heavily dependent on axonal vesicle release (Hines et al., 2015). Moreover, neuronal activity also influences the length of the sheaths (Hines et al., 2015) and the number of myelin sheaths each oligodendrocyte makes (Mensch et al., 2015). It is worth mentioning that to visualize oligodendrocyte lineage cells in different stages, those studies generated transgenic lines that express fluorescence protein under promoters (e.g., Sox10) that are widely expressed at almost all stages within oligodendrocyte lineage. In addition, they often chose to express membrane-anchored fluorescence protein instead of cytoplasm-expressing ones for the better visualization of myelin sheaths. Using synaptic protein-expressing transgenic zebrafish models, several studies further demonstrated the relationship between axonal vesicle exocytosis and myelin sheath formation through *in vivo* time-lapse imaging (Hughes and Appel, 2019; Almeida et al., 2021). To investigate how oligodendrocyte lineage cells react to axonal activity or vesicle release, recent studies also used genetically encoded Ca^2+^ indicators and showed that various myelin sheaths movements are accompanied by Ca^2+^ transients with different duration and frequencies within oligodendrocyte processes (Baraban et al., 2018; Krasnow et al., 2018; Hughes and Appel, 2020). By combining Ca^2+^ imaging with the single-cell RNA sequencing technique, Marisca et al. (2020) also demonstrated that subgroups of OPCs exhibited widespread heterogeneity in eliciting Ca^2+^ responses and initiating proliferation or differentiation upon neuronal activities.

The zebrafish larva as the animal model clearly shows the advantage of *in vivo* imaging due to its transparency. When looking at imaging techniques that can be applied *in vivo* for mouse models, the multi-photon laser scanning system surpasses the confocal system as it penetrates deeper into the tissue with less scattering and phototoxicity. However, it is worth mentioning that 2-photon microscopy generally does not reach more than 500 μm deep in the brain. Imaging deeper brain structures often involves tissue removal and the embedment of a gradient index lens (Jung et al., 2004). Future studies employing adaptive optical correction (Wang et al., 2015) or utilizing 3-photon *in vivo* imaging (Horton et al., 2013; Hontani et al., 2021) might partially overcome this limitation. Recent studies utilizing *in vivo* 2-photon imaging demonstrated how OPCs maintain hemostasis (Hughes et al., 2013), exhibit experience-dependent oligodendrogenesis and myelin adaptation (Hughes et al., 2018), and undergo adaptive remodeling during motor learning activities (Bacmeister et al., 2020). Choosing the appropriate time interval between the consecutive acquisition sessions is critical for these studies because the interval represents the temporal resolution. One must carefully set this parameter to obtain sufficient details about the target cellular event without generating a vast number of images. For example, we now know that OPCs are highly motile and can translocate to a new position within 2–3 days, even in adulthood (Hughes et al., 2013). Therefore, the interval between imaging sessions needs to be set much shorter than 2–3 days to follow the same group of cells over the whole imaging period. Longitudinal *in vivo* imaging studies take over days to weeks and involve re-anesthetizing the animal and re-identifying the exact imaging area between imaging sessions. Setting the imaging area with easily identified landmarks, e.g., visible blood vessels, is often helpful. Imaging processing and analysis are also challenging for *in vivo* time-lapse imaging studies, and it usually requires algorithms to correct moving artifacts and align all images taken at different time points. Due to technical difficulties such as motion artifacts caused by heartbeat and breathing, fewer studies employed time-lapse *in vivo* imaging for the spinal cord than the brain. However, the development of implantable spinal cord windows (Farrar et al., 2012; Tedeschi et al., 2016) may allow future studies to follow the dynamic communication between axons and oligodendroglial cells within the murine spinal cord. Miniaturized microscopes, also called miniscopes, can be directly head-mounted in freely moving animals and allow optical recording over an extended period (Ghosh et al., 2011). However, the conventional miniscope has lower optical resolution than the 2-photon scanning system and therefore cannot resolve finer cellular structures like dendritic spines (Silva, 2017). The ongoing development of 2-photon miniscopes (Zong et al., 2017) might overcome this problem and provide *in vivo* imaging in freely behaving animals with greater optical resolution. Miniscope platforms might be suited for future studies investigating longitudinal changes in oligodendroglial plasticity and myelin formation in various behavioral paradigms.

Besides imaging oligodendroglial cells and myelin sheaths with genetically labeled fluorescence proteins, a couple of label-free *in vivo* myelin imaging techniques have also been developed during the last decade. Coherent anti-Stokes Raman scattering (CARS) microscopy utilizes the intrinsic CH2 symmetric stretching vibration of the enriched lipid structure to image myelin sheaths (Wang et al., 2005; Fu et al., 2008). Meanwhile, spectral confocal reflectance microscopy (SCoRe; Schain et al., 2014) generates label-free myelin images by capturing the highly reflective signals from lipids using several lasers with different wavelengths. In combination with *in vivo* fluorescence microscopy (Schain et al., 2014), those novel label-free techniques can image dynamics between axons and myelin sheaths with great resolution and reduce the dependence on genetic models for *in vivo* imaging of myelin. The long-term stability and phototoxicity of these label-free imaging techniques still need to be characterized for time-lapse *in vivo* imaging studies spanning multiple days.

## Concluding Remarks

We have now reached a widely accepted notion that neuronal activity positively regulates myelin development and adaptation during adulthood. It is clear that activity-dependent myelination is a fairly dynamic process, and future studies will require the design of specific experimental paradigms to capture the dynamics with great temporal and spatial precision. Future studies using a multidisciplinary approach addressing whether inputs from just a few synapses are sufficient to initialize the expression and translation of myelin-specific mRNAs within OPC processes and whether there is any preferred neuronal firing pattern for initiating downstream cellular events will enhance our understanding of the functional role of neuron-OPC synapses. Additionally, monitoring oligodendroglial cellular behavior and tracing key intracellular signaling molecule’s activity *in vivo* in various behavioral paradigms will offer further insights into the mechanisms that control activity-dependent myelination.

## Author Contributions

JH and WS wrote the manuscript. WS conceived the project and supervised the overall progress. All authors contributed to the article and approved the submitted version.

## Conflict of Interest

The authors declare that the research was conducted in the absence of any commercial or financial relationships that could be construed as a potential conflict of interest.

## Publisher’s Note

All claims expressed in this article are solely those of the authors and do not necessarily represent those of their affiliated organizations, or those of the publisher, the editors and the reviewers. Any product that may be evaluated in this article, or claim that may be made by its manufacturer, is not guaranteed or endorsed by the publisher.
